# Title Changes in the Mineral Composition of Rat Femoral Bones Induced by Implantation of LNCaP Prostate Cancer Cells and Dietary Supplementation

**DOI:** 10.3390/nu13010100

**Published:** 2020-12-30

**Authors:** Dorota Skrajnowska, Agata Jagielska, Anna Ruszczyńska, Barbara Wagner, Wojciech Bielecki, Barbara Bobrowska-Korczak

**Affiliations:** 1 Faculty of Pharmacy with the Laboratory Medicine Division, Department of Bromatology, Medical University of Warsaw, Banacha 1, 02-097 Warsaw, Poland; dorota.skrajnowska@wum.edu.pl; 2 Biological and Chemical Research Centre, Faculty of Chemistry, University of Warsaw, Zwirki i Wigury 101, 02-089 Warsaw, Poland; ajagielska@chem.uw.edu.pl (A.J.); aruszcz@chem.uw.edu.pl (A.R.); barbog@chem.uw.edu.pl (B.W.); 3 Department of Pathology and Veterinary Diagnostics, Faculty of Veterinary Medicine, Warsaw University of Live Sciences, Nowoursynowska 159c, 02-787 Warsaw, Poland; wojciech_bielecki@sggw.pl

**Keywords:** supplementation with minerals, bone mineral loss, LNCaP implantation in rats, cancer

## Abstract

Prostate cancer (PCa) is the second most frequent cancer in men and the fifth most common cause of death worldwide, with an estimated 378,553 deaths in 2020. Prostate cancer shows a strong tendency to form metastatic foci in the bones. A number of interactions between cancer cells attacking bones and cells of the bone matrix lead to destruction of the bone and growth of the tumour. The last few decades have seen increased interest in the precise role of minerals in human health and disease. Tumour cells accumulate various minerals that promote their intensive growth. Bone, as a storehouse of elements, can be a valuable source of them for the growing tumour. There are also reports suggesting that the presence of some tumours, e.g., of the breast, can adversely affect bone structure even in the absence of metastasis to this organ. This paper presents the effect of chronic dietary intake of calcium, iron and zinc, administered in doses corresponding maximally to twice their level in a standard diet, on homeostasis of selected elements (Ca, K, Zn, Fe, Cu, Sr, Ni, Co, Mn and Mo) in the femoral bones of healthy rats and rats with implanted cancer cells of the LNCaP line. The experiment was conducted over 90 days. After the adaptation period, the animals were randomly divided into four dietary groups: standard diet and supplementation with Zn, Fe and Ca. Every dietary group was divided into experimental group (with implanted cancer cells) and control group (without implanted cancer cells). The cancer cells (LnCaP) were implanted intraperitoneally in the amount 1 × 10^6^ to the rats at day 90 of their lifetime. Bone tissue was dried and treated with microwave-assisted mineral digestation. Total elemental content was quantified by ICP-MS. Student’s *t*-test and Anova or Kruskal–Wallis tests were applied in order to compare treatment and dietary groups. In the case of most of the diets, especially the standard diet, the femoral bones of rats with implanted LNCaP cells showed a clear downward trend in the content of the elements tested, which may be indicative of slow osteolysis taking place in the bone tissue. In the group of rats receiving the standard diet, there were significant reductions in the content of Mo (by 83%), Ca (25%), Co (22%), Mn (13%), K (13%) and Sr (9%) in the bone tissue of rats with implanted LNCaP cells in comparison with the control group receiving the same diet but without LNCaP implantation. Supplementation of the rat diet with calcium, zinc and iron decreased the frequency of these changes relative to the standard diet, which may indicate that the diet had an inhibitory effect on bone resorption in conditions of LNCaP implantation. The principal component analysis (PCA) score plot confirms the pronounced effect of implanted LNCaP cells and the standard diet on bone composition. At the same time, supplementation with calcium, zinc and iron seems to improve bone composition. The microelements that most often underwent quantitative changes in the experimental conditions were cobalt, manganese and molybdenum.

## 1. Introduction

Despite enormous advances in diagnostics and treatment, it is estimated that even 30–50 years from now, cancer will be the leading cause of death worldwide [1]. Tumour metastasis is believed to be a significant cause of patient mortality [2]. Most often, irrespective of the type of tumour, the cancer metastasizes to the bones, destroying their structure via a variety of mechanisms [2]. Communication between tumour cells attacking the bone and cells building the bone matrix and the nerve fibres innervating them plays a fundamental role in bone metastasis. Prostate cancer cells, similarly to breast cancer cells, have special characteristics conducive to anchoring in the bone marrow [3]. Metastatic cancer cells, besides releasing numerous paracrine factors, e.g., parathyroid hormone-related peptide (PTHrP) and interleukin 6 (IL-6), also affect the immune system, vascular endothelium, nerve cells, and platelets [4,5]. They also stimulate angiogenesis, which conditions their nutrition and survival, as well as the pathological growth of nerve fibres, which is one of the causes of the neuropathic pain accompanying bone metastasis [6,7,8]. The complex interactions between cancer cells attacking the bones and cells of the bone matrix lead to progressive destruction of the bone and to growth of the tumour. In the case of bone tissue, invasion of tumour cells causes an imbalance between the activity of osteoblasts and osteoclasts [9]. A key mechanism regulating the function of cells of the bone matrix is the molecular triad osteoprotegerin (OPG)/receptor activator of NF-kB (RANK)/RANK ligand (RANKL) pathway [10]; disturbance of this mechanism by cancer cells leads to increased osteolysis [8,11,12]. Furthermore, bone structure can be influenced by aggressive treatments that destroy cancerous cells and tissues, while disturbing the function of healthy ones. Adverse effects are also found in the endocrine system and organs. In general, mineral imbalance and changes in the skeletal system lead to secondary forms of osteopenia or osteoporosis in patients of all ages [13]. The varied effect (positive or negative) of mineral supplementation on the course of cancer should also be considered, especially given that many elements play an important role in both maintenance of homeostasis and the course of cancers, including PCa. These elements unquestionably include calcium, zinc and iron. The role of zinc in bone tissue is twofold. First, it is a component of the bone matrix, so it performs a structural function. Hydroxyapatite contains ions of this element, mainly in the form of complexes with fluorine and phosphates [14]. Secondly, zinc takes an active part in bone formation by stimulating osteoblasts and inhibiting bone resorption by osteoclasts [11,15]. This type of effect is possible mainly because zinc ions are components of enzymes, i.e., alkaline phosphatase, carbonic anhydrase, and DNA and RNA polymerase, and also activate aminoacyl-tRNA synthetase in the osteoblasts, stimulating protein synthesis and bone formation processes [11,15,16]. Zinc has been shown to protect against loss of bone mass in rats [17]. Moreover, the bone tissue of osteoporosis patients has been shown to have reduced content of this microelement [18,19,20]. In the urine of post-menopausal women, zinc can be a marker of bone resorption, as patients with osteoporosis excrete much larger quantities of this element [18,21]. Furthermore, epidemiological research has shown that middle-aged and older people whose diet is low in zinc are more susceptible to bone fractures [19,21]. Apart from its effect on bones, zinc plays an important role in prostate function [22]. Dysfunctional zinc metabolism in the prostate gland is associated with a variety of disturbances, and above all can be evidence of prostate cancer [23,24,25]. The primary function of zinc in the prostate is to prevent oxidation of citrate in the Krebs cycle by inhibiting mitochondrial activity of m-aconitase [26]. An inadequate zinc concentration in the prostate probably leads to excessive oxidation of citrate and increased ATP production. An elevated ATP level results in excessive proliferation and the risk of DNA damage, including neoplastic transformation [26]. Changes in zinc content in prostate tissues are also linked to changes in testosterone metabolism. Zinc is regarded as an androgen modulator [27]. It takes part in regulation of the transformation of testosterone into the biologically active compound 5α-dihydrotestosterone by 5α-reductase. The dihydrotestosterone–cytoplasmic receptor complex is transported to the prostate cell nucleus, where it induces RNA transcription and synthesis of receptor and secretory proteins of the prostate [27,28,29]. Due to the major role of zinc in prostate cells, it has been suggested that dietary supplementation with zinc can be used in the prevention and treatment of prostate cancer [30]. On the other hand, results on the effectiveness of zinc supplementation can be largely dependent on the dose and duration of supplementation [30,31]. No increase in the risk of prostate cancer has been observed in men taking zinc supplements at doses not exceeding 100 mg/day. However, in men taking zinc in doses above 100 mg/day for 10 years or more, the risk of advanced prostate cancer was doubled [30]. This is because an excess of zinc enhances telomerase activity, which is already increased in prostate cancer, leading to excessive proliferation of tumour cells [32]. Moreover, an excess of zinc in prostate cells antagonizes the potentially inhibitory effect of biophosphates on the metastasis of cancer cells [33]. Moreover, high zinc intake is linked to the level of circulating IGF-1 (insulin-like growth factor) and testosterone, factors increasing the risk of prostate cancer [34]. High zinc intake (over 150 mg/24 h) also exerts adverse metabolic effects, such as immune dysfunction and damage to the antioxidant system, which are potentially associated with the risk of prostate cancer [35,36]. Therefore, both high and low zinc concentrations in the prostate tissues can probably increase the risk of cancer.

The second element discussed is iron. Like copper, iron catalyses oxidation, and thus, in excessive amounts, leads to an increase in reactive oxygen species, which can generate lipid oxidation and degrade other high-molecular-weight compounds (including DNA), thereby contributing to damage, mutation or death of the cell [37,38,39]. Iron has also been found to promote the development of the disease after its onset, because by functioning as a nutrient for cancer cells it promotes their growth and increases inflammation. Moreover, it has been shown to take part in angiogenesis and thereby foster tumour development [39,40,41]. The results of research on the existence of a relationship between cancer development and iron are inconclusive. Some studies link an increased risk of disease to elevated levels of haemoglobin, plasma iron or transferrin (TF), while others have found neither a link nor the reverse relationship [37]. The discrepancies in research results may be due to inherent difficulties in conducting the analyses, such as deductive methods of assessing iron intake, differences in the degree of absorption, or genetic polymorphism [42,43]. One study confirmed higher iron content in human prostate cancer cells than in healthy ones, with the concentration increasing with the advancement of the disease [39]. However, the values obtained for different groups were highly varied, so that iron assays could not be used to distinguish between stages of cancer. It was determined that elevated content of this element could increase the risk of cancer or be a consequence of it, given the tumour’s increased demand for trace elements [39]. Cancer cells increase the amount of bioavailable iron not only by increasing its absorption and decreasing the amount stored, but also by reducing its efflux. One of the most important recent discoveries is the existence of the hepcidin–ferroportin axis. These two proteins play an important role in regulating the level of iron in the body and in carcinogenesis [37]. Both an excess and a deficiency of iron in the body are linked to bone weakening, because iron influences the differentiation and activity of osteoclasts and osteoblasts [12]. Excess iron causes osteoclast differentiation and activation, which leads to destruction of the bone. Excess iron impairs osteogenic differentiation of mesenchymal stem cells (MSC) and the functions of mature osteoblasts, causing bone resorption by osteoclasts and reducing bone formation by osteoblasts [12,44,45]. Many studies indicate a positive correlation between the development of osteoporosis and excess iron [12,46].

Calcium is the best known skeletal component, with 99% of its total pool in the body deposited in bone tissue in the form of hydroxyapatite and other calcium salts [47]. This element, as the primary mineral component of the bone matrix, determines the hardness and strength of its tissue [47]. The most common symptom of bone tumour lysis is the loss of calcium from the bone mass. This is often accompanied by hypercalcaemia [48]. It has also been demonstrated that disturbances of cell cycle control due to differences in calcium ion concentrations can be crucial in neoplastic transformation [49,50]. Many epidemiological studies have assessed the link between a diet containing calcium and the risk of various cancers, including prostate cancer [51,52,53,54,55]. The literature contains a great deal of data on the role of calcium and dairy products in neoplastic transformation of the prostate [54,55,56]. The problem is that the data (in contrast to the data on colorectal cancer) are inconsistent [49,57]. There are many reasons for these discrepancies, such as assessment of the effect of dairy products as a whole (without taking into account the type or fat level) or failure to consider factors such as the degree of advancement of the disease, when the test was conducted (before or after diagnosis), concomitant diet, or hormonal disorders. There are several hypotheses attempting to explain the link between increased intake of calcium with dairy products and the risk of prostate cancer. The most commonly mentioned mechanism of the effect of calcium on the development of prostate cancer is the reduced concentration of 1,25-dihydroxycholecalcypherol (1,25(OH)_2_D_3_) circulating in the blood due to excess dietary intake of this element. On the other hand, dietary supplementation with calcium is sometimes seen as beneficial for the prognosis of colorectal cancer [58,59,60]. Tumour cells accumulate various minerals, which, due to their fundamental role in biochemical processes, can also facilitate tumour development [61]. Bone, as a large storehouse of elements, can be an important source of them for the growing tumour. As mentioned above, PCa often forms metastatic foci in the bones, leading to destruction of bone tissue. There are also reports that the presence of tumours, e.g., in the breast, can negatively affect bone structure even if there is no metastasis to this organ. This effect has been demonstrated by Thorpe et al. [62], who conducted research on female rats with mammary cancer induced by N-methyl-N-nitrosourea (MNU). Despite the absence of metastasis, the femoral bones of rats with induced mammary cancer had lower mineral density and mechanical strength and poorer structural parameters than the control group. Furthermore, a strong inverse relationship was shown between the total mammary tumour mass and the quality characteristics of the bone tissue. These results suggest that some interactions between tumour cells and the bone microenvironment may occur even in the absence of metastasis.

Our paper presents the effect of supplementation of the diet of rats with minerals that play an important role in prostate function. The study investigated the effect of chronic intake of calcium, zinc, and iron in doses corresponding maximally to twice their level in a standard diet on homeostasis of selected elements in the femoral bones of healthy rats and rats with implanted tumour cells of the LNCaP line. The aim of the study was to determine the effect of implantation of LNCaP cells on the mineral composition of the femoral bone of rats in the early stage of tumour development and to establish whether enriching the diet with various minerals can significantly modify the mineral composition of the bone in conditions of the implantation of prostate cancer cells.

## 2. Materials and Methods

### 2.1. Ethics Approval Statement

This research and guiding principles in care and use of laboratory animals were approved by IInd Local Ethical Committee on Animal Experiments at the Medical University of Warsaw.

### 2.2. Dietary Ingredients

Labofeed H feed, used in our research, is intended for adult rats and provides them with optimal conditions for growth and reproduction. Laboratory fodder Labofeed H was purchased from the “Morawski” Feed and Concentrates Production Plant (Kcynia, Poland). The contents of selected minerals in animal feed are presented in Table 1.

The diet contained the following other compounds (per 1 kg): protein (210 g), fat (39.2 g), fiber (43.2 g), ash (55 g), carbohydrates (300 g), vitamin A (15,000 IU), vitamin D3 (1000 IU), vitamin E (90 mg), vitamin K3 (3 mg), vitamin B1 (21 mg), vitamin B2 (16 mg), vitamin B6 (17 mg), vitamin B12 (80 μg), pantothenic acid (30 mg), folic acid (5 mg), nicotinic acid (133 mg), P (8.17 g), Mg (3 g), K (9.4 g), Na (2.2 g), Cl (2.5 g), S (1.9 g), Mn (100 mg), Cu (21.3 mg), Co (2.0 mg), I (1.0 mg), and Se (0.5 mg).

### 2.3. Animal Experiment

Male Sprague-Dawley rats (n = 54) were obtained from the Animal Laboratory, Department of General and Experimental Pathology from the Medical University of Warsaw. The rats were kept under the standard conditions of the animal house with 12-h light–dark cycle at a temperature 22 °C. They had free access to food (standard diet: Labofeed H) and water.

The experiment was conducted over 90 days. After the adaptation period (10 days—rats’ age 60 to 70 days), the animals were randomly divided into four dietary groups: standard diet and supplementation with Zn, Fe, Ca. Every dietary group was divided into experimental group (with implanted cancer cells) and control group (without implanted cancer cells). The cancer cells (LnCaP) were implanted intraperitoneally in the amount of 1 × 10^6^ (in PBS 0.4 mL) to the rats at day 90 of their lifetime. The certified line of androgen-dependent human prostate cancer cells was obtained from ATTC bank (American Type Culture Collection, Menassas, VA, USA).

**Figure 1:**
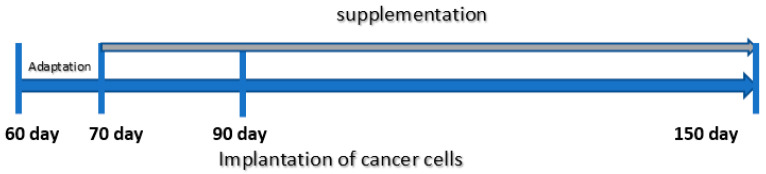
Scheme of the experimental procedure.

The rats were fed extra supplements suspended in water, 0.4 mL daily, from 70 days until 150 days of age, when they were sacrificed by decapitation (Figure 1). The animals that were fed only the standard diet (without supplementation) received 0.4 mL of water. The doses of trace elements were selected based on the values used in the Labofeed H diet (extrapolated on the rats’ body weight). According to the level of trace elements in the Labofeed diet, the rats were fed, via gavage, extra supplements of the following: double dose of Zn, or one dose of Fe or a quarter dose of Ca. The doses of selected minerals were chosen based on their levels in dietary supplements which are commonly used by people and can be available at any pharmacy.

The animals from both groups—experimental (Exp) and control (Ctrl)—were provided with the minerals by oral gavage in a solution:

- Zinc 4.6 mg/mL (1.85 mg Zn (II)/day/rat) (as ZnSO_4_ × 7H_2_O in aqueous suspension);
- Iron 7.5 mg/mL (3 mg Fe (II)/day/rat) (as FeSO_4_ × 7H_2_O in aqueous suspension);
- Calcium 75 mg/mL (30 mg Ca/day/rat) (as CaCl_2_ × 6H_2_O in aqueous suspension).

Additional dietary supplementation in particular groups resulted in the following total intakes: 500 mg Fe, 230.7 mg Zn and 12.5 g Ca/kg feed.

The material for the study was rat femoral bones. Following resection, the adjacent soft tissues, i.e., the joint capsule and muscle, were removed from the bones, and then they were frozen at −80 °C. Immediately before analysis, the samples were thawed, dried for 10 h at 120 °C, and mineralized in 5 mL 65% HNO3 solution (Plazmatronika mineralizer). Then deionized water was added to the digest solution to a volume of 10 mL. The conditions for each stage of mineralization are presented in Table 2.

The content of ten elements (Ca, K, Fe, Sr, Zn, Ni, Cu, Mn, Co and Mo) was determined in the digest solutions by inductively coupled plasma mass spectrometry (ICP-MS), using the following dilutions:

(1) Five-fold for determination of Co, Cu, Mn, Mo, Ni, Se and Zn;
(2) 500-fold for determination of Ca, K, Sr and Fe.

#### 2.3.1. Chemicals and Reagents

Stock solutions were prepared by diluting ICP multi-element standard Merck VI (Merck, Germany). Samples and standards were diluted with deionized water obtained by the Milli-Q System (Merck, Millipore, Germany).

#### 2.3.2. Instrumentation

Isotope-specific detection was achieved using a quadrupole mass spectrometer with inductively coupled plasma ionization, ICP-MS, (Nexion 300D, Perkin Elmer, Boston, MA USA) equipped in a quartz cyclonic spray chamber and Meinhard nebulizer. The working conditions of the spectrometer were optimized daily in order to obtain the maximal sensitivity and stability as well as the lowest level of oxides and double-charged ions (Table 3).

#### 2.3.3. Analytical Procedure

Quantitation was achieved by five-point external calibration (standards from 1 to 100 μg·L^−1^ for K, Mn, Co, Cu, Ni, Sr, Mo; standards from 10 to 1000 μg·L^−1^ for Fe, Se and Zn; standards from 100 to 10,000 μg·L^−1^ for Ca).

### 2.4. Histopathology

The rats were sacrificed by decapitation at 150 days of age, and tumours were evaluated histopathologically. The tissues were recorded in a buffered formalin solution, then they were dehydrated, sealed in paraffin and cut into scraps 4 µm thick. The hematoxylin and eosin staining of tissue and cell sections was applied, and the sections were evaluated using a BX43 Olympus research microscope.

### 2.5. Statistics

Student’s *t*-test was used to compare the content of individual elements in the control vs. experimental groups, separately for each diet. Differences between groups were considered significant at *p* < 0.05. Statistical analysis was performed with the use of Metaboanalyst 4.0 platform and R (RStudio ver. 1.2.5019). the obtained data were log-transformed, and their normality was assessed based on the Shapiro–Wilk (SW) test and qq plots, and then the homoscedasticity using Bartlett’s test, respectively. To test differences in concentrations of metals between analyzed groups, the ANOVA or Kruskal–Wallis (KW) tests were used; for ANOVA or KW significant results (q-value <0.05), Tukey HSD or Dunn post-hoc tests were performed subsequently; all *p*-values were corrected. In some cases, the Principal Components Analysis (PCA) was performed for the data overview (data were auto scaled). For the graphical data representation, dendrograms were also prepared, based on the Euclidean distance method.

## 3. Results

### 3.1. Characteristics of the Animals’ Body Weight Gain

The animals’ body weight gain is presented in Table 4. There were no statistically significant differences between the increase in the body weight of the rats with intraperitoneally implanted prostate cancer cells compared to the non-implanted rats obtaining the same supplementation. As concerns the rats that were not implanted with cancer cells, a statistically higher increase in body weight was found in the animals whose diet was supplemented with iron (124.7 ± 10.6), as compared to those that received only the standard diet (100.2 ± 11.7) (Table 4).

### 3.2. Mean Values with Standard Deviations for the Content of Ten Elements in Bone of Rats

Mean values with standard deviations for the content of 10 elements in the bone of rats are presented in Table 5, separately for different diet groups and for different treatment groups: Exp (experimental with implanted cancer cells LNCaP) and Ctrl (control without implanted cells LNCaP).

### 3.3. Experimental to Control Group Comparison

Elemental content ratios between experimental and control groups are presented in Table 6.

The experimental-to-control ratios (%) for the content of elements indicate a downward trend in most cases. The statistically significant ratio (Ctrl/Exp) calculated for the standard group and the group receiving the zinc supplement is less than one. The reverse tendency was noted only in the group receiving an iron supplement, in which the content of certain elements increased in the bones of rats with LNCaP implantation (Table 6). An especially large number of differences in the mineral composition of the bones between the groups with and without implanted LnCaP were generated by the standard diet (without supplementation). In the group of rats receiving this diet, there were significant reductions in the content of Mo (by 83%), Ca (25%), Co (22%), Mn (13%), K (13%) and Sr (9%) in the bone tissue of rats with implanted LNCaP cells in comparison with the control group receiving the same diet but without LNCaP implantation. There were no changes in the concentration of Zn, Fe, Cu or Ni, but all analyzed elements showed a decreasing trend (Table 5 and Table 6).

In the group of rats receiving a diet supplemented with zinc, there was a statistically significant reduction in Cu (by 38%), Co (36%) and Mn (20%) in the bone tissue of rats with implanted LNCaP cells in comparison to the control group that also received zinc supplementation (Table 5 and Table 6).

Slightly different results were obtained in the case of the diet supplemented with iron; there was a small but statistically significant increase in the content of Zn (by 9%) and Co (8%) and a strong increase in Mn (by 32%), as well as an increasing tendency for most of the elements (except Ca and Ni).

In the group of rats receiving a diet supplemented with calcium, no statistically significant differences were noted in the mineral composition of the bones between the groups with implanted LnCaP cells and the control group on the same diet (Table 5 and Table 6).

### 3.4. Comparison of Diet Groups

#### 3.4.1. Dietary Supplementation with Calcium Relative to Other Experimental Groups

Multi-factor ANOVA test or Kruskal–Wallis (KW) tests were performed to compare different dietary groups

**Figure 2:**
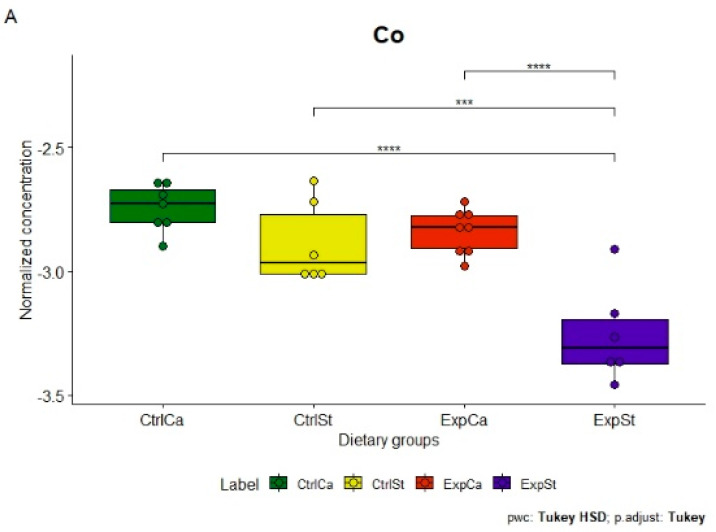

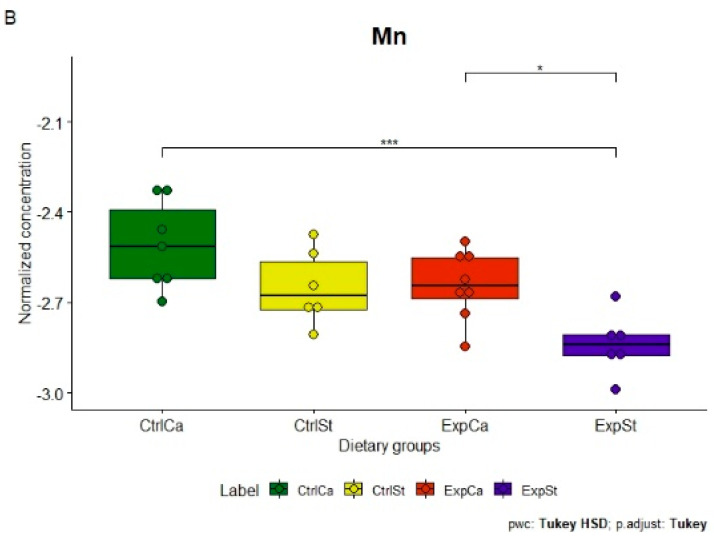

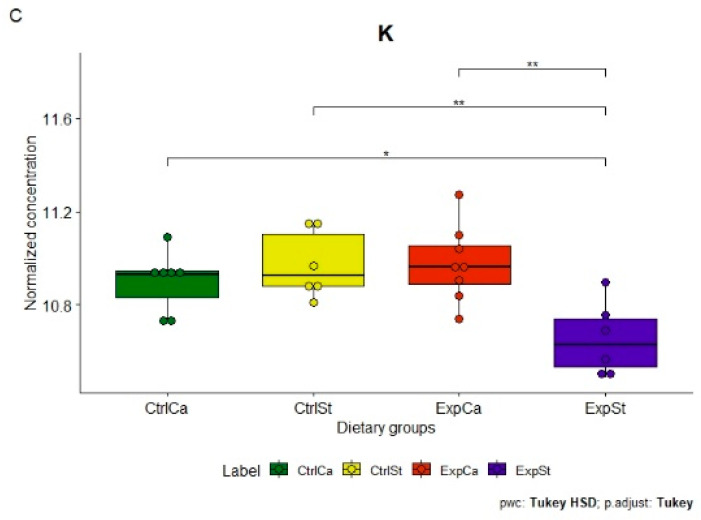

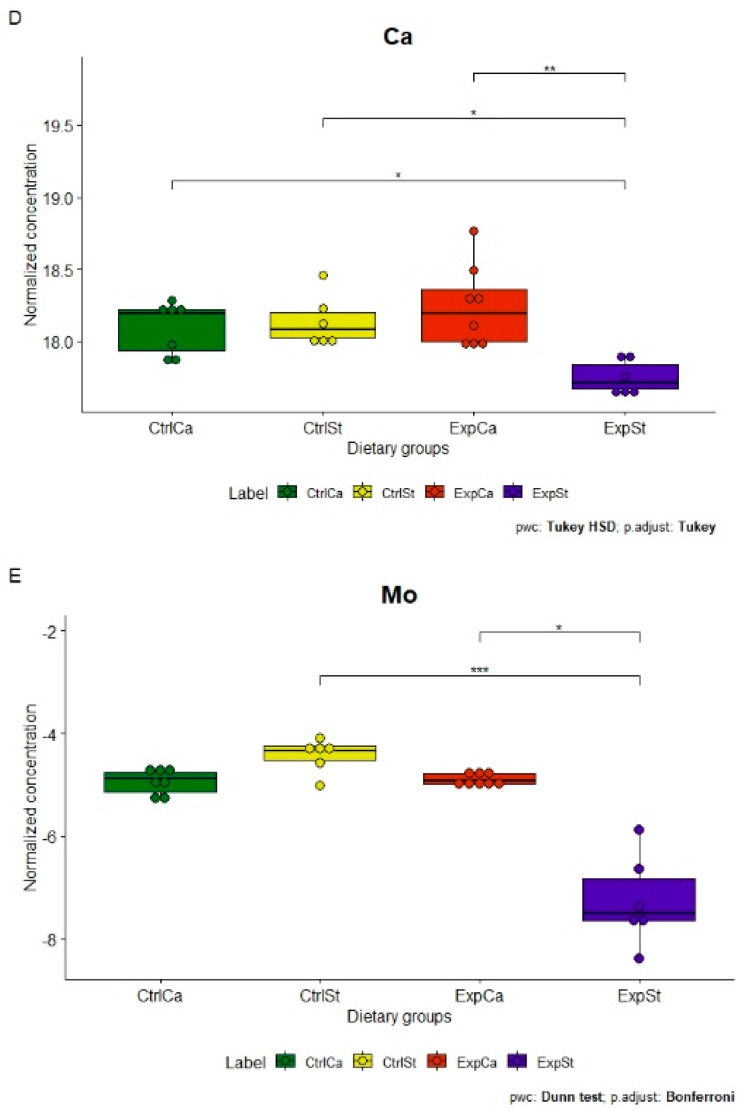
Analysis of concentrations of elements in four experimental groups: ExpSt—experimental standard diet with LNCaP (violet boxplot), CtrlSt—control standard diet without LNCaP (yellow boxplot); ExpZn—Zn supplementation diet with LNCaP (green boxplot); CtrlZn—Zn supplementation diet without LNCaP (red boxplot); *p*-value: <0.0001 ****, 0.0001–0.001 ***, 0.001–0.01 **, 0.01–0.05 *.

**Figure 3:**
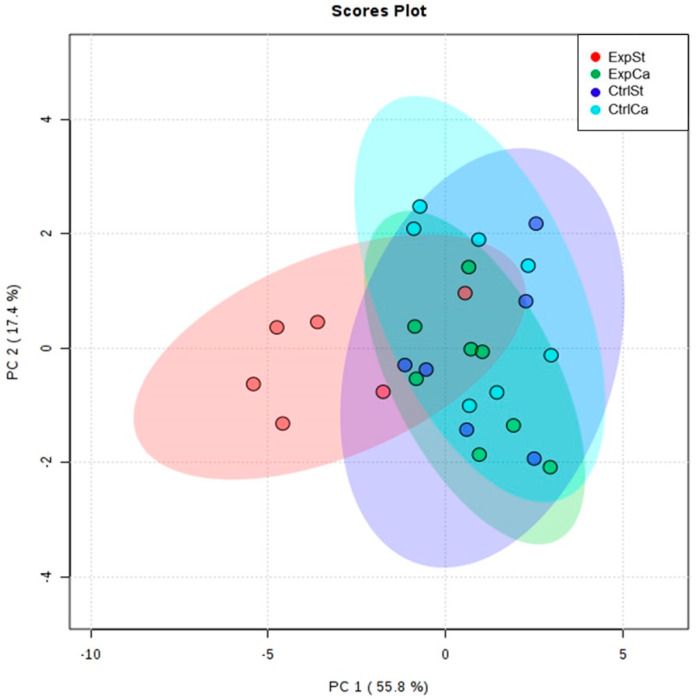
The Principal Component Analysis performed on four groups—rats on the standard diet with LNCaP (red dots—ExpSt); rats on the standard diet without LNCaP (blue dots—CtrlSt); rats with Ca supplementation with LNCaP (green dots—ExpCa); rats with Ca supplementation without LNCaP (light blue dots—CtrlCa).

The following statistically significant relationships were obtained (Figure 2):

- Supplementation of the diet of rats with calcium ions, irrespective of LNCaP implantation (CtrlCa and ExpCa), caused a significant increase in the content of Co, Mn, K and Ca in comparison to the group with implanted LNCaP on the standard diet (ExpSt) (Figure 2A–D). In the case of Mo, such a relationship was noted only for the group with implanted LNCaP cells (ExpCa) (Figure 2E);
- Supplementation of the diet of rats with calcium ions in the groups with implanted LNCaP (ExpCa) caused no differences in the level of elements in relation to the other groups (CtrlCa and CtrlSt) (Figure 2A–E);
- Implantation of LNCaP cancer cells in the group on the standard diet (ExpSt) significantly decreased the level of Co, K, Ca, Mo and Mn in the bones (Figure 2A–E);
- No significant differences were noted in the case of the other elements tested (Fe, Zn, Sr, Cu and Ni; data not shown);
- The PCA (principal component analysis) score plot confirms the pronounced effect of implanted LNCaP cells and the standard diet on bone composition (ExpSt is separated from the other groups) (Figure 3). At the same time, supplementation with calcium seems to improve bone composition (similarity of ExpCa to CtrlSt and CtrlCa).

#### 3.4.2. Dietary Supplementation with Zinc Relative to Other Experimental Groups

**Figure 4:**
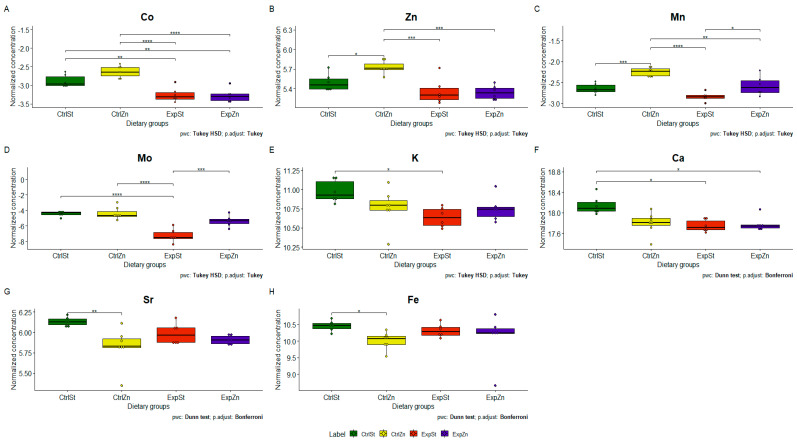
Analysis of concentrations of elements in four experimental groups: ExpSt—experimental standard diet with LNCaP (violet boxplot), CtrlSt—control standard diet without LNCaP (yellow boxplot); ExpZn—Zn supplementation diet with LNCaP (green boxplot); CtrlZn—Zn supplementation diet without LNCaP (red boxplot); *p*-value: <0.0001 ****, 0.0001–0.001 ***, 0.001–0.01 **, 0.01–0.05 *.

**Figure 5:**
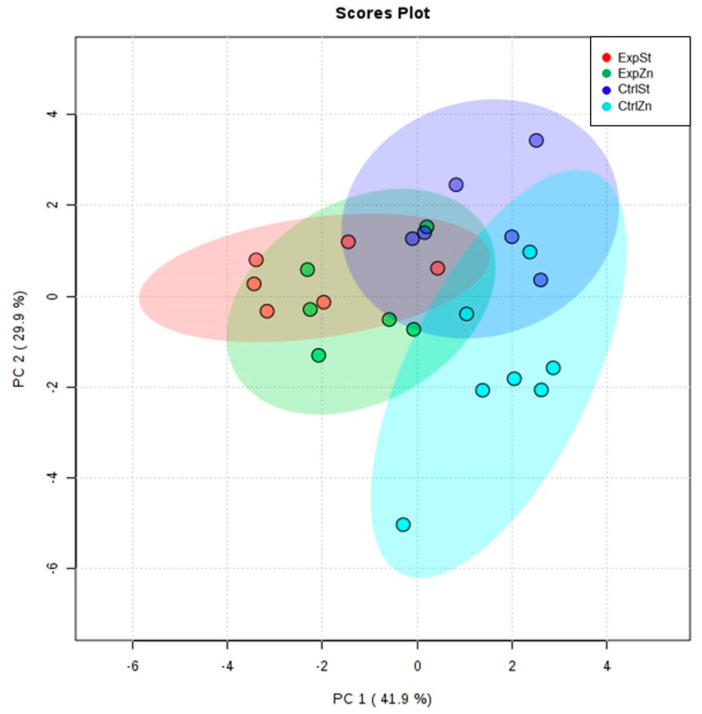
The Principal Component Analysis performed on four groups—rats on the standard diet with LNCaP (red dots—ExpSt); rats on the standard diet without LNCaP (blue dots—CtrlSt); rats with Zn supplementation with LNCaP (green dots—ExpZn); rats with Zn supplementation without LNCaP (light blue dots—CtrlZn).

The following statistically significant relationships were obtained (Figure 4):

- Dietary supplementation with zinc generated the most differences between groups in the case of Co, Mn, Mo and Zn (Figure 4A–D);
- Dietary supplementation with zinc in the control groups without implantation of LNCaP (CtrlZn) significantly increased the content of Zn, Mn and Co in the bones relative to both groups with implanted LNCaP (ExpZn and ExpSt) (Figure 4A–C), but for Mo only in relation to ExpSt (Figure 4D);
- The content of Sr and Fe was reduced in the control groups receiving a zinc supplement (CtrlZn) relative to the standard control group (CtrlSt) (Figure 4G–H);
- Implantation of LNCaP cells in groups ExpSt and ExpZn reduced the level of Ca relative to the standard control group (CtrlSt) (Figure 4F);
- No significant differences were noted in the case of Cu or Ni (data not shown);
- Principal component analysis shows a clear influence of dietary supplementation with zinc in rats without LNCaP implantation on bone composition (CtrlZn is separated from the other groups). At the same time, LNCaP implantation causes changes in bone composition (similarity of ExpZn to ExpSt) (Figure 5).

#### 3.4.3. Dietary Supplementation with Iron Relative to Other Experimental Groups

**Figure 6:**
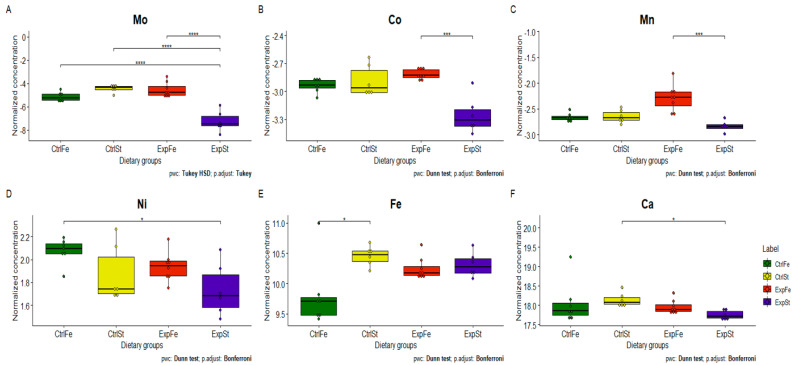
Analysis of concentrations of elements in four experimental groups: ExpSt—experimental standard diet with LNCaP (violet boxplot), CtrlSt—control standard diet without LNCaP (yellow boxplot); ExpFe—Fe supplementation diet with LNCaP (green boxplot); CtrlFe—Fe supplementation diet without LNCaP (red boxplot); *p*-value: <0.0001 ****, 0.0001–0.001 ***, 0.01–0.05 *.

**Figure 7:**
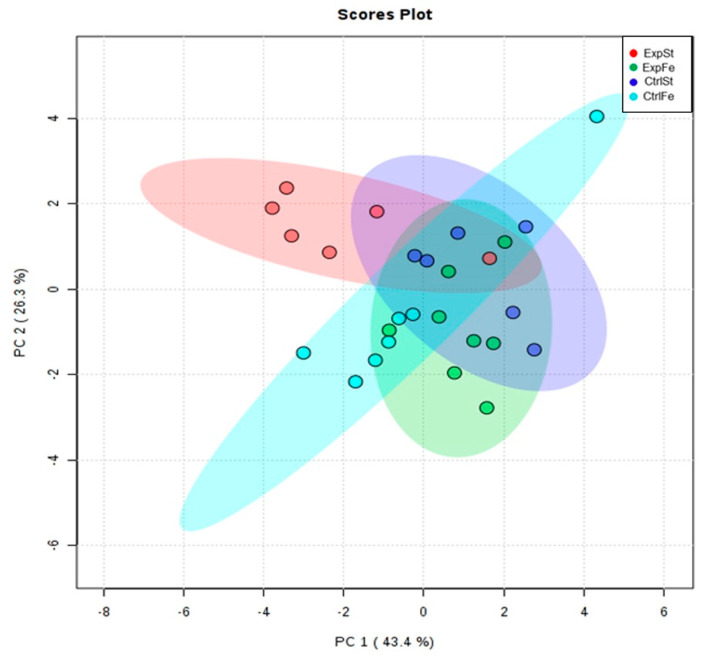
The Principal Component Analysis performed on four groups—rats on the standard diet with LNCaP (red dots—ExpSt); rats on the standard diet without LNCaP (blue dots—CtrlSt); rats with Fe supplementation with LNCaP (green dots—ExpFe); rats with Fe supplementation without LNCaP (light blue dots—CtrlFe).

The following statistically significant relationships were obtained (Figure 6):

- The Mo level in the groups whose diet was supplemented with iron, irrespective of the presence or absence of LNCaP cells (CtrlFe and ExpFe), as well as in group CtrlSt, was significantly higher than in the standard group with implanted LNCaP (ExpSt) (Figure 6A);
- Implantation of cells of the LNCaP line in the group on an iron diet (ExpFe) significantly increased the content of Co and Mn in the bones relative to the experimental group on the standard diet (ExpSt) (Figure 6B–C);
- In the control group on the iron diet (CtrlFe), there was an increase in the content of Ni (relative to ExpSt) (Figure 6D) and a decrease in Fe (relative to CtrlSt) (Figure 6E);
- The calcium concentration in the femoral bone of rats in the implantation groups (ExpSt) was lower than in the groups without implanted cells on the standard diet (CtrlSt) (Figure 6F);
- There were no changes in the case of Cu, Zn, Sr or K (data not shown);
- Principal component analysis indicates that the presence of cancer cells had a marked effect on the bone composition of rats on the standard diet (ExplSt is separated from the other groups). At the same time, iron supplementation improved bone composition (similarity of ExpFe to CtrlSt and CtrlFe) (Figure 7).

### 3.5. The Effect of Supplementation with Calcium, Iron and Zinc on the Occurrence of Tumour Hyperplasia in Rats

**Figure 8:**
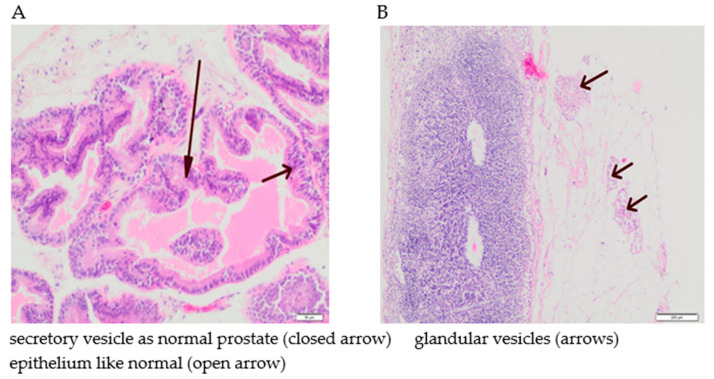

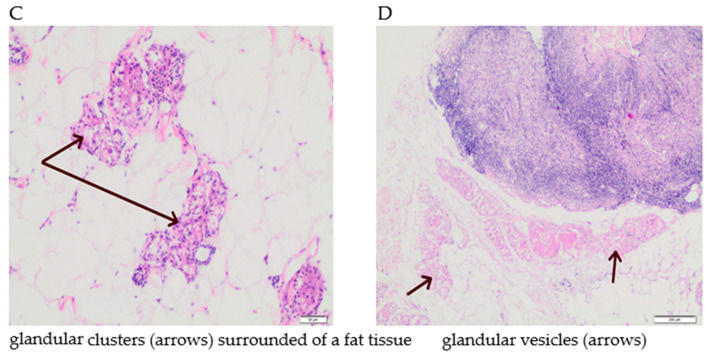
Hematoxylin-eosin-stained sections of anterior prostate from rats implanted LNCaP cells. (**A**)—rats fed standard diet—without supplementation; (**B**)—rats fed diet supplemented with iron; (**C**)—rats fed diet supplemented with calcium; (**D**)—rats fed diet supplemented with zinc.

The tumours were located subcutaneously, mainly in lymph nodes. The greatest proliferation of tumour cells and the greatest number of their aggregates in lymph nodes were found in the rats whose diet was supplemented with iron (100%). The histopathological studies showed that the cells in the epithelium displayed prostate gland cell characteristics, with the possibility of tumour growth, but with no signs of inflammation (Figure 8). The cells were characterized by a benign phenotype with no features of prostate cancer. No deferent ducts were also found. In non-implanted groups, spontaneous cancers were not observed.

## 4. Discussion

Several key findings emerge from the present study. First, implantation with prostate cancer cells of the LNCaP line significantly influenced the mineral composition of the bones. This was particularly pronounced in the group receiving a diet without mineral supplementation (standard diet), in which the concentration of most tested elements decreased (Mo, Co, Ca, Mn, K and Sr). Secondly, enrichment of the diet of rats with calcium, zinc or iron modified the mineral composition of the bone tissue to varying degrees but reduced the frequency of changes relative to the standard diet. Third, while the effect of calcium [19,63,64,65,66], zinc [67,68,69,70] and iron [71,72] on bone condition is relatively well documented, the role of the trace elements Mo, Co and Mn in bone physiology, especially in cancer, is not. However, it is the concentrations of these three bioelements that most often underwent changes following implantation of LNCaP, irrespective of dietary supplementation. Their effect on skeletal metabolism can be twofold: indirect, through regulation of metabolism of macroelements, or direct, through their effect on the proliferation or activity of osteoblasts or osteoclasts, or finally by incorporation into the bone mineral matrix. PCa often forms metastatic foci in the bones, leading to the destruction of bone tissue. In our study, we did not observe fractures or changes in bone structure or mechanical strength. It is possible that before the onset of metastasis (specific bone colonization), prostate cancer cells release factors stimulating bone tissue resorption. In this way, the tumour obtains nutrients that promote its growth, which at the same time increases the susceptibility of bone tissue to future invasion by the tumour. Appropriate dietary supplementation might help to minimize the risk of loss of certain minerals from the bone tissue. Bone is a metabolically active tissue that reacts to changes in the diet and nutrition. It consists primarily of an organic layer formed by collagen fibres, proteoglycans, some lipids, and proteins (35%). The inorganic part of the bone structure is mainly calcium phosphate (about 80%), calcium carbonate, citrates, ions of magnesium sodium, potassium, and fluorine, and also small amounts of boron, selenium, copper, silicon, strontium, molybdenum, manganese, cobalt and others, including toxic elements such as lead and cadmium [11,73].

The doses of elements used in our study to enrich the diet were maximally twice their levels in feed; they caused no toxic symptoms in the animals during the entire experiment. Furthermore, no significant differences were observed in weight gain or in the weight of the liver or spleen, induced by either dietary supplementation or implantation of cancer cells. In our previous study, we also described the effectiveness of implantation of LNCaP cancer cells as the number of tumours and disease incidence among rats [74]. On the standard diet, tumours appeared in 71% of animals, on the calcium-enriched diet in 88%, and on the zinc-enriched diet in 50%. There were no cancerous changes in the rats fed these diets in the control groups, without implanted LNCaP. We concluded that the implantation of LNCaP cells caused tumour induction. Thus, despite the lack of visible morphological changes in the femoral bones, normal mobility in the rats, and the absence of pain symptoms, we tested the mineral composition of the bone tissue. Metastasis to the bone is a common complication of cancer development and causes pain, pathological fractures and hypercalcaemia [75,76]. During cancer, local stimulation of the activity and recruitment of osteoclasts takes place in the bone. Cytokines produced by cancer cells increase production of proteolytic enzymes in the osteoclasts, which leads to local bone lysis and the release primarily of calcium [75,76].

The elemental composition of femoral bones presented in this study can be compiled with the results obtained for prostate gland of the same animals [77]. The same effect of LNCaP implantation on calcium content was observed for standard diet group (0.8 ExpSt:CtrlSt ratio, statistically significant in both cases). Additionally, for strontium a statistically significant reduction in the content was noticed after LNCaP implantation for both tissues from standard diet group. This may suggest similar effects on two organs by pathological process.

It should be emphasized that the dietary supplementation in our study essentially did not cause changes in the content of calcium, zinc or iron in the bones. As mentioned above, the implantation of cancer cells of the LNCaP line in the group of experimental rats significantly modified mainly the levels of Mo, Co and Mn. How do these elements affect bone condition and what are the potential future consequences of their deficiency?

Manganese is essential for the body, primarily as a cofactor of enzymes taking part in the hydrolysis, phosphorylation, decarboxylation and transamination of many compounds. It is responsible for the metabolism of nutrients such as amino acids, cholesterol and carbohydrates, supports the immune system and blood sugar balance, and takes part in cellular energy production, reproduction and bone growth [78]. It determines the activity of transferases such as glycosyltransferase, glutamine synthetase and superoxide dismutase [79]. Manganese deficiency does not normally occur in humans due to low requirements and the availability of food products containing manganese. There have also been no reported cases of toxicity in humans due to manganese intake in the diet. The adverse effect on the central nervous system of mine and steel mill workers is well documented. [78]. Experimentally induced Mn deficiency in rats and mice causes poor growth, infertility, and bone deformation [78]. This underscores the important role of manganese as a factor contributing to the formation of bone cartilage and bone collagen, as well as in bone mineralization [19]. Mn is known to take part as a cofactor in the formation of chondroitin sulphate through its effect on two enzymes: polymerase and galactosyltransferase [80]. This is essential for building the structure of hyaline cartilage. Thus, an Mn deficiency will significantly impair the quality of the organic bone matrix due to the insufficient amount of chondroitin sulphate [81,82]. Bone, as the primary tissue storing manganese (43%), is one of the main organs used to assess the deposition of Mn in the body and the overall health benefit or risk to the body associated with this element [83]. However, little is known of the rate of accumulation of Mn in human or animal bone or of the biological half-life of Mn. Researchers have estimated Mn t_1/2_ in the entire rat skeleton, and, after averaging the values for all bones, determined that following chronic oral exposure to Mn t_1/2_, is about 143 days [84,85]. Taking into account the body weight of humans and rats, Sengupta et al. [86] proposed that 16.7 days of the life of a rat was equivalent to one year of human life, so that the average half-life of Mn in the rat skeleton corresponds to 8.6 years for humans. Importantly, Mn levels in the bone were correlated with Mn levels in the striatum, hippocampus and CSF, which indicates that the Mn level in the bone is a useful biomarker of exposure to Mn, especially given that classic matrices such as blood and urine are not suitable for biomonitoring [83]. A weak link has been shown between Mn concentrations in the blood and urine and levels of external exposure [87]. Moreover, the half-life of Mn in the blood is short (less than 2 h) [88] and plasma concentrations of Mn measured in a study of chronic exposure fell after two weeks, although exposure to Mn was still ongoing [83]. It should also be stressed that over 95% of Mn is excreted with bile into the faeces, so its concentration in the urine is very low. It is possible to analyse manganese in samples of human saliva, but due to the fairly high variation in the results, this is not recommended for an evaluation of exposure to this element [84,89]. The data indicate that bone is a good biomarker for assessing both exposure to Mn and its status in the body [90].

O’Neal et al. [82] observed no significant changes in the body weight of animals following chronic oral exposure to Mn, but femoral bone weight fell after 18 weeks of exposure. Thus, apart from the storage function, Mn ions accumulated in the bones can have a direct harmful effect on bone structure and function. Mn was also found to be positively correlated with Fe and Zn in the bones, but inversely correlated with Cu. The consequences of these relationships are unknown; it was concluded that osteotoxicity induced by Mn due to chronic exposure in humans and animals should be further studied, especially since accumulation of Mn in the bone probably affects other essential elements in this tissue. On the other hand, manganese deficiency is linked to osteoporosis and congenital skeletal disorders, including chondrodystrophy [91,92,93,94,95].

Other studies on rats have shown that a diet rich in manganese prevents cartilage formation and induces osteopenia, due in part to an imbalance between osteoblast and osteoclast activity. Human studies have shown that osteoporosis patients have low serum levels of manganese. In another randomized two-year study, dietary supplementation with manganese, copper, zinc and calcium was more effective than calcium supplementation alone in preventing loss of bone density [19]. A study with induced Fe deficiency in rats showed an increase in absorption of intestinal Mn [96]. Mn is particularly beneficial in osteosis and synthesis of mucopolysaccharides in the cartilage of young rats [97]. Furthermore, in some studies Mn deficiency disturbed levels of hormones involved in bone metabolism and induced a reduction in bone markers in chicken serum [98]. There was also an interesting study indicating osteoporosis as a new risk factor for uterine cancer [99]. According to the authors, appropriate concentrations of antioxidant elements such as zinc, copper and manganese can be helpful in prevention and treatment of osteoporosis, and subsequently in reducing the risk of uterine cancer.

In our study, implantation of LNCaP line cancer cells caused a significant loss of manganese in the bones of rats fed a standard diet. The cancer cells seem to have been the main factor stimulating this unfavourable process. Dietary supplementation with calcium, zinc and iron in our study, despite partially modifying the manganese level in the femoral bone of the rats, to some degree, reduced the scale of this phenomenon observed in the case of the standard diet without supplementation. The changes observed in the Mn levels may be positively correlated with future deterioration of bone density. Both a suitable diet and moderate aerobic exercise are known to protect the bones and cartilage, most likely in an antiradical mechanism involving trace elements taking part in the biosynthesis of structures of the bone matrix and inhibition of bone resorption. In healthy individuals, stable manganese levels in the tissues are maintained by strict homeostatic control of the intestines, involving the absorption and excretion of bile. The neoplastic process, even in the early stages and located far from the bone tissue, can activate destruction of the bone tissue, which may first be manifested as a loss of manganese from the bone tissue. The data obtained suggest that LNCaP implantation in rats disturbs mechanisms of Mn homeostasis.

Another interesting trace element is molybdenum. The changes it undergoes seem to be closely dependent on the diet used and implantation. In the group of rats receiving the classic standard diet, its content in the femoral bone of rats exposed to LNCaP fell dramatically by over 80%. In contrast, the zinc diet for the control and the iron diet for both groups significantly increased the concentration of molybdenum in the femoral bone of the rats relative to the experimental group receiving the standard diet.

Molybdenum is an essential trace element for nearly all organisms [100,101]. It is found in human tissues in a range from 0.001 to 0.4 mg/kg of tissue, with the lowest values in the blood and the highest in the kidneys and liver [73,102]. The average value in the soft tissues is <0.075 mg/kg and, in the skeleton, <0.48 mg/kg [103]. Deficiencies of this element are rarely observed in humans, but a lack of or inadequate activity of enzymes containing Mo may have serious consequences, e.g., mental retardation [100]. Enzymes containing Mo are redox-active and catalyse metabolic reactions in nitrogen, sulphur and carbon cycles [104]. In order to obtain biological activity and perform its function in enzymes, Mo must be complexed by the compound pterin, thus forming the molybdenum cofactor Moco [105]. Biosynthesis of Moco involves a complex interaction of six proteins and is a multi-stage process dependent on iron, ATP and copper. After synthesis, Moco is distributed to apoproteins of Mo-enzymes by Moco-carrier/binding proteins [105,106]. Moco deficiency due to inadequate biosynthesis or a metabolic defect causes serious neurodegenerative changes in infants and early death in childhood [100,105,106]. This is probably due to a pleiotropic loss of activity of all human molybdenum enzymes [106]. Four Mo-dependent enzymes have been identified in humans: sulphite oxidase (detoxification of sulphites, i.e., oxidation of sulphite to sulphate and degradation of sulphur-containing amino acids), xanthine oxidoreductase (degradation of purines; oxidation of hypoxanthine to xanthine and xanthine to uric acid), aldehyde oxidase (degradation of heterocyclic compounds and aldehydes, purine, and pteridine) and mitochondrial reductase (activation of prodrugs containing an amidoxime structure and metabolism of N-hydroxylated purine and pyrimidine bases [107,108,109,110]. It should be noted that the physiological role of enzymes containing Mo is largely unknown [104], and there is a lack of studies evaluating the relationship between biomarkers of exposure to Mo and bone condition [101]. Analysis of bone mineral density (BMD) is a non-invasive test of bone condition. BMD is closely linked to the peak bone mass attained during adolescence and its later loss with age in both sexes, which can lead to osteoporosis and the risk of fractures [111]. The number of osteoporosis cases was predicted to rise by nearly 20% by 2020, negatively affecting quality of life through increased chronic pain and disability [112,113].

The mechanism by which molybdenum affects BMD is unknown; it is believed that it may be an indirect effect, through disturbance of the serum testosterone level [101]. R.C. Lewis et al. [101] attempted to show the effect of Mo on androgen secretion by testing the relationship between the Mo concentration in the urine and BMD. The role of sex steroid hormones in maintaining bone density is well known [114]. The role of oestrogen in stimulating bone growth and maturation and inhibiting bone resorption is particularly well documented [115]. In men, androgens influence bone metabolism and are essential for the promotion of skeletal integrity [116]. Testosterone reduces bone resorption (possibly via aromatization of oestrogen) and improves bone formation [117]. In men with hypogonadism, in which the testosterone level is very low, BMD is significantly decreased [117] and long-term androgen therapy increases BMD irrespective of ageing, which indicates the role of testosterone in bone formation [117,118]. Numerous studies have shown that Mo can alter the serum testosterone level [109,119] and thus influences BMD in adults.

The biological mechanisms by which Mo can affect BMD are not fully understood, but it is likely that this trace element may disturb the level of sex steroid hormones essential for bone health [114]. Oestrogen directly affects bone cells and is of fundamental importance for skeletal growth and bone homeostasis in both men and women [120]. In an in vitro study, Mo induced a substantial dose-dependent decrease in the release of oestrogen in fragments of rat ovaries [109]. Androgens also affect skeletal health, though to a lesser extent than oestrogen, through direct interactions with bone cell receptors [121], stimulating longitudinal bone growth and decreasing apoptosis in osteocytes [121]. In our study, implanted LNCaP cells are characterized by the presence of androgen receptors on their surface. Treatment with androgen antagonists currently plays a fundamental role in advanced prostate cancer treatment, mainly by reducing androgen production through surgical or chemical castration; the search for innovative antagonists among structural analogues is ongoing [122]. In light of the literature data cited and our own results, further research is needed to confirm the specific potential effect of Mo on the endocrine system, not only in terms of its effect on BMD, but also regarding its involvement in tumour development. Was the molybdenum released from the bones of rats on the standard diet in our study a factor causing hormonal destabilization, promoting further development of the tumour, or was it preparation for colonization of the bone tissue by cancer cells, or perhaps both? Did the iron-enriched diet significantly increase the molybdenum concentration in the femoral bone of rats relative to the experimental group on the standard diet due to the close link between molybdenum and iron associated with Moco biosynthesis and the functioning of most Mo enzymes in higher organisms? The answers to these questions require further, comprehensive research. Our study is limited due to the lack of sex hormone levels (particularly testosterone) in the blood serum and elements involved in the metabolism of the active form of molybdenum (Moco)—iron and copper [123].

The final trace element whose concentration in the bone is significantly decreased by LNCaP and a standard and zinc-enriched diet, and slightly decreased by an iron-enriched diet, is cobalt. According to current knowledge, cobalt is an essential microelement for animals and humans. It is a key component of vitamin B12 (cobalamin) and plays an important role in the formation of amino acids and certain proteins of nerve cell myelin, and thus in the formation of neurotransmitters essential for the functioning of the body [124]. A cobalamin deficiency in the blood serum (<130 pg/mL) is associated with anaemia, including megaloblastic anaemia, and degenerative changes in the gastric mucosa [125]. The role of cobalt in bone tissue function is not fully understood and is perceived mainly in terms of the risks associated with the use of orthopaedic implants (in use for over 40 years), in which cobalt and its alloys are major components. The angiogenic capacity of cobalt ions gave rise to the idea of including this metal in various materials used for bone healing, in order to stimulate the vascularization of implanted materials and thus improve the reconstruction process and support overall regeneration [82,126]. However, postoperative complications caused by contact allergy to cobalt or metallosis are a serious medical problem that can result in rejection of the transplant [124,126]. Metallosis refers to the local effect of metal ions or products of corrosion of the implant on tissues. The complications it causes involve destruction of the soft tissues around the prosthesis, osteolysis, pseudotumours, and infiltrates composed of lymphocytes and plasmatic cells during an immune reaction to a given metal [127,128]. Cobalt metallosis, i.e., poisoning by cobalt released from a prosthesis, can be referred to as cobaltosis. Inorganic, ionized forms of cobalt are toxic for humans (unlike organic forms, which are biodegradable), and the longer the exposure, the greater the changes in the cells [124]. A high Co level (from 10 to 1000 times the norm) in the blood of patients with implants containing this element has been described in many studies. Apart from destruction of the bone surrounding the prosthesis, it can exert systemic effects whose clinical significance are still unclear [125,128,129]. In vitro studies using animal cells have shown that cobalt and chromium ions exceeding physiological concentrations induce dose-dependent apoptosis in human osteoblasts and inhibit the synthesis of osteoblasts [130,131,132]. Bone metabolism is defined by the interaction between osteoclasts, which are responsible for bone resorption, and osteoblasts, which ensure bone formation. The predominance of osteoclast activity induced by various external factors, such as systemic diseases and endocrine disorders, as well as nutrition, and accelerate bone loss and increase the risk of fractures [133]. In another study, higher Co concentrations (>10 mg/mL) affected osteoblasts in two ways: first by decreasing the proliferation and synthesis of osteoblasts and osteocalcin, and secondly by increasing the production of IL-6. Suggested mechanisms of the toxic effect of cobalt ions may involve radical formation, impairment of cell membrane function, or inhibition of enzyme function [132].

Did the release of cobalt in our study, most likely induced by the LNCaP cells, translate into a systemic effect, or was it only the beginning of the destruction of bone tissue? Unfortunately, we did not test cobalt levels in the blood, which might have helped to answer that question. Undoubtedly, however, the homeostasis of trace elements in the bone tissue in experimental conditions was disturbed. Due to a number of inconsistencies in the scientific literature regarding the role of trace elements in the bone tissue and the conviction that the composition of this tissue is constant, further research is needed to confirm the effect of various diseases, including prostate cancer, various doses of elements, and dietary supplementation with macro- and micro-elements on bone health and treatment. Although there have been many interesting results in rat studies, we are aware that there are limitations concerning human biology. Therefore, orthotopic xenografts of transplanted cancer cell lines would be a better model to analyze metastatic prostate cancer, including its metastases to the bone.

## Figures and Tables

**Table 1:** Mineral content in Labofeed H feed.

Element	Element Concentration in Feed (Per 1 kg)
Ca	10.0 g
Zn	76.9 mg
Fe	250.0 mg

**Table 2:** Stages and conditions of mineralization.

Step	Time (min)	Power (%)	Minimum Pressure (atm)	Maximum Pressure (atm)
1	1	70	17	20
2	5	80	27	30
3	6	90	36	39

**Table 3:** Instrument conditions.

ICP-MS Nexion 300D	
RF power	1350 W
nebulizer gas flow (Ar)	0.9 l/min
dwell time	50 ms
readings	5
sweeps	1
replicates	3
monitored isotopes	^24^Mg, ^27^Al, ^39^K, ^43^Ca, ^51^V, ^55^Mn, ^57^Fe, ^59^Co, ^63^Cu, ^60^Ni, ^66^Zn, ^78^Se, ^88^Sr, ^95^Mo, ^111^Cd, ^208^Pb

**Table 4:** The animals’ body weight gain [g] (week 10–21).

Diets	The Animals’ Body Weight Gain (g) (Week 10–21)
ExpSt	117.4 ± 31.2
ExpZn (4.6 mg Zn/mL)	101.8 ± 11.0
ExpCa (75 mg Ca/mL)	102.4 ± 11.6
ExpFe (7.5 mg Fe/mL)	120.6 ± 12.2
CtrlSt	100.2 ± 11.7 *
CtrlZn (4.6 mg Zn/mL)	113.1 ± 14.9
CtrlCa (75 mg Ca/mL)	94.8 ± 8.5
CtrlFe (7.5 mg Fe/mL)	124.7 ± 10.6 *

ExpSt—experimental standard diet with LNCaP, CtrlSt—control standard diet without LNCaP; ExpZn—experimental zinc-supplemented diet with LNCaP, CtrlZn—control zinc-supplemented diet without LNCaP ExpCa—experimental calcium-supplemented diet with LNCa; CtrlCa—control calcium-supplemented diet without LNCaP; ExpFe—experimental iron-supplemented diet with LNCaP; CtrFe—control iron-supplemented diet without LNCaP; *—groups of control rats; group fed standard diet versus groups fed diet with addition of trace elements (*p* < 0.05).

**Table 5:** Mean values ± standard deviation of the elemental content (mg/kg, dry mass of bone) in controls and experimental groups of rats (content of calcium—g/kg).

Elements ↓	CtrlSt ExpSt (n = 12)	CtrlZn ExpZn (n = 14)	CtrlFe ExpFe (n = 14)	CtrlCa ExpCa (n = 14)
Zn	45.3 ± 4.3	53.0 ± 3.6	40.9 ± 2.0	45.3 ± 2.7
	41.3 ± 6.0	40.6 ± 3.1	44.5 ± 2.9	43.2 ± 1.4
Fe	1411 ± 158	1040 ± 176	961 ± 482	1371 ± 228
	1281 ± 184	1197 ± 449	1224 ± 169	1381 ± 118
Ca	291 ± 38	230 ± 32	293 ± 148	280 ± 34
	221 ± 18	226 ± 24	256 ± 33	315 ± 67
Cu	0.494 ± 0.261	0.386 ± 0.03	0.273 ± 0,013	0.343 ± 0.026
	0.272 ± 0.084	0.241 ± 0.04	0.305 ± 0.044	0.312 ± 0.013
Mn	0.160 ± 0.014	0.211 ± 0.015	0.158 ± 0.009	0.176 ± 0.018
	0.140 ± 0.010	0.169 ± 0.031	0.208 ± 0.039	0.160 ± 0.012
Co	0.136 ± 0.016	0.162 ± 0.018	0.131 ± 0.007	0.150 ± 0.01
	0.106 ± 0.015	0.104 ±0.014	0.142 ± 0.005	0.141 ± 0.008
Mo	0.047 ± 0.010	0.056 ± 0.036	0.029 ± 0.008	0.033 ± 0.006
	0.008 ± 0.005	0.027 ± 0.015	0.047 ± 0.024	0.035 ± 0.002
K	2018 ± 206	1758 ± 281	1756 ± 631	1918 ± 169
	1753 ± 484	1732 ± 205	2027 ± 255	2028 ± 236
Sr	70.1 ± 2.7	57.2 ± 8.6	67.4 ± 25	67.8 ± 7.3
	63.5 ± 5.8	62.2 ± 6.6	64.2 ± 7.01	72.1 ± 6.8
Ni	3.71 ± 0.69	4.19 ± 0.53	4.22 ± 0.31	3.91 ± 0.28
	3.36 ± 0.55	6.60 ± 7.06	3.83 ± 0.35	3.77 ± 0.25

ExpSt—experimental standard diet with LNCaP, CtrlSt—control standard diet without LNCaP; ExpZn—experimental zinc-supplemented diet with LNCaP, CtrlZn—control zinc-supplemented diet without LNCaP ExpCa—experimental calcium-supplemented diet with LNCa; CtrlCa—control calcium-supplemented diet without LNCaP; ExpFe—experimental iron-supplemented diet with LNCaP; CtrFe—control iron-supplemented diet without LNCaP; n- total of number rats.

**Table 6:** Direction and percentage of changes in the elements in the bone of rats with cancer receiving various diets as compared to the control group on the same diet. Statistically significant results are bolded.

Elements		ExpSt:CtrlSt	ExpZn:CtrlZn	ExpFe:CtrlFe	ExpCa:CtrlCa
Zn		↓9%	↓23%	↑9%	↓5%
	*p* value	ns	ns	0.013	ns
Fe		↓9%	↑15%	↑27%	↑1%
	*p* value	ns	ns	ns	ns
Ca		**↓24%**	↓2%	↓13%	↑13%
	*p* value	**0.002**	ns	ns	ns
Cu		↓45%	**↓38%**	↑12%	↓9%
	*p* value	ns	**0.0001**	ns	ns
Mn		**↓13%**	**↓20%**	**↑32%**	↓9%
	*p* value	**0.017**	**0.011**	**0.005**	ns
Co		**↓22%**	**↓36%**	**↑8%**	↓6%
	*p* value	**0.007**	**0.0001**	**0.003**	ns
Mo		**↓83%**	↓48%	↑62%	↑6%
	*p* value	**0.0001**	ns	ns	ns
K		**↓13%**	↓2%	↑15%	↑6%
	*p* value	**0.002**	ns	ns	ns
Sr		**↓9%**	↑9%	↓5%	↑6%
	*p* value	**0.029**	ns	ns	ns
Ni		↓9%	↑58%	↓9%	↓4%
	*p* value	ns	ns	ns	ns

ExpSt—experimental standard diet with LNCaP, CtrlSt—control standard diet without LNCaP; ExpZn—experimental zinc-supplemented diet with LNCaP, CtrlZn—control zinc-supplemented diet without LNCaP ExpCa—experimental calcium-supplemented diet with LNCa; CtrlCa—control calcium-supplemented diet without LNCaP; ExpFe—experimental iron-supplemented diet with LNCaP; CtrFe—control iron-supplemented diet without LNCaP; ↓—decrease; ↑— increase; *p*-value ≤ 0.05 was considered significant.
